# Data for action – description of the automated COVID-19 surveillance system in Denmark and lessons learnt, January 2020 to June 2024

**DOI:** 10.1017/S0950268825000263

**Published:** 2025-03-14

**Authors:** Gudrun Witteveen-Freidl, Karina Lauenborg Møller, Marianne Voldstedlund, Sophie Gubbels, Statens Serum Institut COVID-19 Automated Surveillance Group

**Affiliations:** 1 Department of Data Integration and Analysis, Infectious Disease Preparedness, Statens Serum Institut, Copenhagen, Denmark; 2 Infectious Disease Epidemiology and Prevention, Infectious Disease Preparedness, Statens Serum Institut, Copenhagen, Denmark; 3 Infectious Disease Preparedness, Statens Serum Institut, Copenhagen, Denmark

**Keywords:** Automation, Systems Integration, Public Health Surveillance, COVID-19, SARS-CoV-2, Denmark

## Abstract

Denmark is one of the leading countries in establishing digital solutions in the health sector. When SARS-CoV-2 arrived in February 2020, a real-time surveillance system could be rapidly built on existing infrastructure, This rapid data integration for COVID-19 surveillance enabled a data-driven response. Here we describe (a) the setup of the automated, real-time surveillance and vaccination monitoring system for COVID-19 in Denmark, including primary stakeholders, data sources, and algorithms, (b) describe outputs for various stakeholders, (c) how outputs were used for action and (d) reflect on challenges and lessons learnt. Outputs were tailored to four main stakeholder groups: four outputs provided direct information to individual citizens, four to complementary systems and researchers, 25 to decision-makers, and 15 informed the public, aiding transparency. Core elements in infrastructure needed for automated surveillance had been in place for more than a decade. The COVID-19 epidemic was a pressure test that allowed us to explore the system’s potential and identify challenges for future pandemic preparedness. The system described here constitutes a model for the future infectious disease surveillance in Denmark. With the current pandemic threat posed by avian influenza viruses, lessons learnt from the COVID-19 pandemic remain topical and relevant.

## Introduction

**Figure 1.**
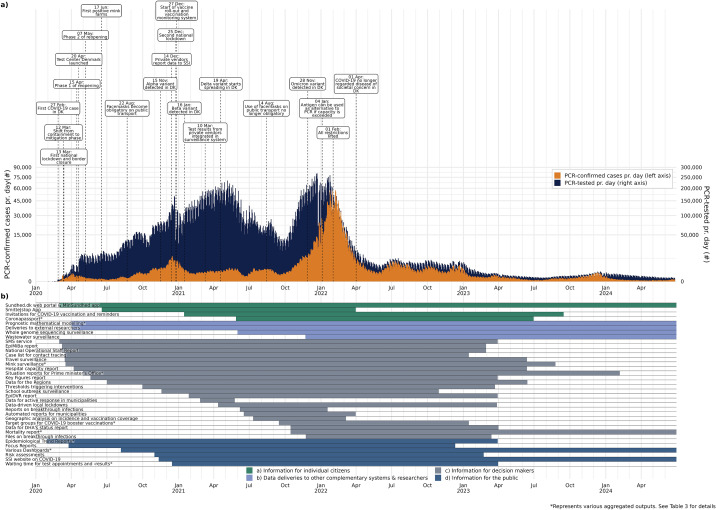
(a) Epidemic curve showing the number of PCR-confirmed COVID-19 cases and the number of persons tested by PCR per day as of January 2020 in Denmark including key events and (b) timespan indicating when outputs were produced in relation to the epidemic. Outputs are coloured according to the four main stakeholder groups as described in Table 3. The number of tests and confirmed cases reflects persons tested by PCR. Antigen tests for SARS-CoV-2 played an important role in the epidemic response in Denmark, with the number of antigen tests performed per day ranging from ~100.000–500.000 between March 2021 and March 2022. Persons testing positive by antigen tests were advised to get confirmation via PCR due to the superior sensitivity and specificity of the latter. Therefore, the majority of outputs are based on PCR-confirmed cases (Table 3).

Infectious disease surveillance allows monitoring trends, describing the epidemiology and burden of disease in a population, enables the early detection of outbreaks, epidemics, and new pathogens, and thereby informs data-driven public health responses [1]. When the new SARS-CoV-2 virus started to emerge in Denmark, with the first case detected on 26 February 2020, Statens Serum Institut (SSI), which is responsible for the national infectious disease preparedness, rapidly set up an automated, real-time surveillance system for COVID-19 within days. Figure 1a shows key milestones of the epidemic in Denmark that unfolded in form of several waves caused by various variants, as well as response measures taken. Two national lockdowns were implemented, and the COVID-19 vaccine roll-out started at the end of December 2020. Denmark’s epidemic response was also characterized by a high test capacity.

In this article, we outline the setup of the automated, real-time integrated surveillance and vaccination monitoring system for COVID-19 in Denmark, describe how outputs for various stakeholders were used for action and reflect on challenges and lessons learnt. This surveillance system also constitutes a model for the future infectious disease surveillance system in Denmark.

## Methods

### Country profile

Denmark is part of the European Union (EU) and has a population of roughly 5.9 million people and a geographical size of 42 925 km^2^ [2].

Every resident of Denmark has free access to the Danish healthcare system and receives a health insurance card that includes their name and civil registration number (CPR number) – a unique personal identifier assigned to every resident of Denmark. The majority of treatments and examinations are free [3].

### Primary stakeholders in the epidemic response in Denmark

Table 1 shows primary stakeholders in the epidemic response and their roles and responsibilities. SSI falls under the Ministry of the Interior and Health and is responsible for the prevention and control of infectious and congenital diseases through research, surveillance, and advice. Since the legal basis for receiving data differed by stakeholder, outputs, calculations, and visualizations needed to be tailored accordingly.

**Table 1.** Primary stakeholders in the epidemic response in Denmark with a focus on roles and responsibilities during the epidemic Indentations and different font types in the stakeholder column reflect the hierarchy among stakeholders

Stakeholder	Roles and responsibilities
Prime Minister’s office	- ultimate responsibility for Denmark’s COVID–19 response
National Operational Staff (NOST)	- country’s crisis management committee that comes together in times of major crises or incidents in Denmark - consists of representatives of the National Police (group leader), Denmark’s National Security and Intelligence Service, the Danish Defence Intelligence Service, the Defence Command, the Danish Emergency Management Agency, the Ministry of Foreign Affairs, the Danish Critical Supply Agency, the Danish Health Authority and the Danish Civil Aviation and Railway Authority (additional authorities can be invited, as needed) - the primary task is to ensure cooperation and coordination of operational efforts across authorities - before, during, and after an incident or emergency, members meet several times a day until returning to normal conditions - particularly active at the very start of the COVID–19 response
Parliament	- to take decisions on the pandemic response based on information received through different channels
Epidemic Committee	- to advise the Minister of the Interior and Health and other ministers, on its own initiative or upon request, on the handling of dangerous diseases of societal concern - facilitated by a representative of the parliament - includes representatives from key stakeholders, e.g. the directors of Statens Serum Institut (SSI) and the Danish Health Authority (DHA)
Ministry of the Interior and Health (MoIH)^a^	- responsible for a wide range of healthcare-related issues - ten institutions fall under this Ministry, of which the five listed below were closely involved in the COVID–19 response:
Danish Health Authority (DHA)	General responsibilities: - (i) to provide advice and support to the Ministry of the Interior and Health, the Danish Regions, municipalities, and the general population on health matters - (ii) to ensure high-quality healthcare on a national level, - (iii) to effectively manage health emergencies and - (iv) to develop guidelines and educational programmes. During the COVID–19 epidemic: - to develop recommendations and guidelines on testing (for confirmed cases and close contacts), vaccination, and infection prevention - to coordinate efforts to maintain sufficient hospital capacity
*Danish National Immunization Technical Advisory Group (NITAG) (‘Vaccinationsråd’)*	- to set out the vaccination strategy - experts in the fields of immunology, infectious disease epidemiology, vaccinology, vaccine side effects, clinical research, and behavioural science. Includes representatives from SSI.
Statens Serum Institut (SSI)	- to prepare for, prevent, and control infectious diseases in Denmark - to establish the automated COVID–19 surveillance - to provide data, which has served as the basis of the data-driven response to the COVID–19 epidemic in Denmark and beyond - to provide epidemiological interpretation of data, to conduct risk assessments, to monitor vaccination coverage, effectiveness and safety, to prepare surveillance reports based on COVID–19 data and conduct research
*Mathematical modelling group*	- to provide the government with prognostic models to facilitate decisions regarding lock-downs and strategies for re-opening - The group was established during the COVID–19 epidemic as an independent entity with members from Statistics Denmark, five Danish universities, and the Niels Bohr Institute. Coordinated by SSI.
Danish Patient Safety Authority (DPSA)	- to trace close contacts of confirmed COVID–19 cases - to train contact tracers - to develop a digital self-service solution for contact tracing in response to the Omicron wave, which started in November 2021 and peaked in early February 2022 with ~57.000 cases per day, when manual contact tracing was no longer possible - provide advice to municipalities, schools, and nursing homes
Danish Health Data Authority (DHDA)	- to provide the authorities under the Ministry of the Interior and Health with a number of registers (e.g. National Patient Register, Cause of Death Register, the Danish Vaccination Register etc.) - to maintain servers - to provide support to SSI in establishing the automated COVID–19 surveillance system and generation of outputs in response to vast numbers of data requests
Danish Medicine Agency (DMA)	- to approve vaccines - to monitor side effects of vaccines in collaboration with SSI
Ministry of Social Affairs and Senior Citizens	From December 2021: - to ensure that COVID–19 recommendations reached vulnerable groups and the elderly (e.g. in nursing homes) - to ensure that close contacts of these groups adhered to the recommendations to protect these groups
Ministry of Children and Education (MoCE)	- responsible for the education of children and adults
Ministry of Defence	- to ensure security and defend the interests of citizens nationally and internationally
Danish Emergency Management Agency (DEMA)	- responsible for emergency management in Denmark and, on demand, also abroad - to set up mobile test centres to increase test capacity when new variants emerged - planning of hospital capacity during COVID–19 - to support Denmark’s COVID–19 test and vaccination centres
The Media and citizens	- to inform the public and to stay informed, thereby supporting community engagement in response measures
Researchers	- to study unknown aspects of a newly emerging virus through register-based research
Citizen scientists	- to conduct private analyses and visualizations of the COVID–19 epidemic
Regions	- the Danish healthcare system is primarily public, universal, and free of charge for citizens - organized in three political and administrative levels: national (state), regional (Regions) and local (municipalities) - the five Danish regions are responsible for this public healthcare system - to secure sufficient hospital capacity, effective treatment, and protection of staff.
Departments of Clinical Microbiology (DCMs)	- ten DCMs are responsible for performing diagnostics of infectious diseases across the five Danish regions and advising hospital departments and general practitioners on hygiene, treatment, and prevention of infectious diseases
Municipalities	- local response, including efforts to increase testing activity in areas with high transmission - responsible for overseeing lockdowns in parishes - to optimize vaccine uptake, including providing support to nursing homes and citizens in booking of vaccination appointments
Danish Critical Supply Agency (DCSA)	- responsible for monitoring capacity and ordering of SARS-CoV–2 tests and personal protective equipment - to ensure that test centres were available within a certain radius of each resident to allow low-threshold access to testing
European Centre for Disease Prevention and Control (ECDC)	- to monitor the evolution of the pandemic in the European Union/European Economic Area - provide guidance and data through situation updates, scientific and technical publications, and risk assessments
World Health Organization (WHO)	- to provide guidance, coordination, and leadership to respond to the pandemic on a global level

a Between 27 June 2019 and 14 December 2022: ‘Ministry of Health and Elderly Affairs’.

### Setup of the Danish COVID-19 surveillance and vaccination monitoring system

**Figure 2.**
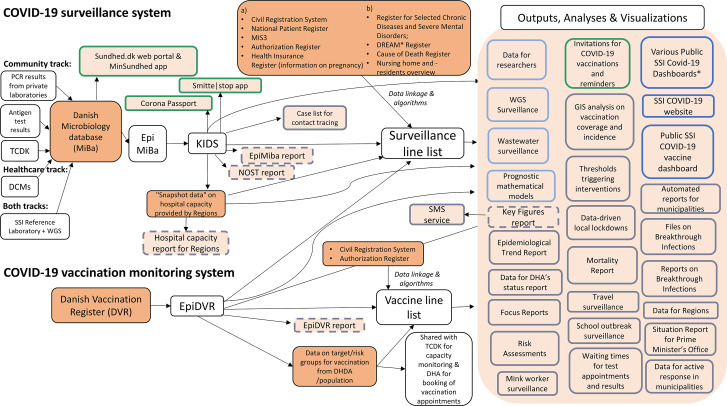
Overview of the dataflow of the Danish COVID-19 surveillance and vaccination monitoring system, including data sources, outputs, analyses, and visualizations. Data sources and registers are depicted in dark orange and outputs, analyses and visualizations in light orange. Column (a) indicates data sources that SSI already had permission for to use for surveillance purposes prior to the COVID-19 epidemic, and column (b) shows existing data sources to which access was granted for surveillance purposes during the COVID-19 epidemic. Coloured outlines indicate the four main stakeholder groups, as described in Table 3, to which outputs, analyses, and visualizations were tailored. In green – information for individual citizens, in light blue – data deliveries to other complementary systems and researchers, in grey – information for decision-makers, and in dark blue – information for the public. Automated e-mail reports are depicted with a dashed outline. Abbreviations: DCMs, Departments of Clinical Microbiology; TCDK, Test Center Denmark; MiBa, Danish Microbiology Database; SSI, Statens Serum Institut; KIDS, Keys to Infectious Disease Surveillance; NOST, National Operational Staff; CRS, Civil Registration System; NPR, National Patient Registry; DHDA, Danish Health Data Authority; DVR, Danish Vaccination Register; DPSA, Danish Patient Safety Authority; DHA, Danish Health Authority; DREAM, database from which type of employment was extracted (see Table 2), WGS, whole genome sequencing.

Figure 2 shows an overview of the dataflow of the Danish COVID-19 surveillance and vaccination monitoring system, including data sources used (Table 2) and outputs produced (Table 3). The following sections describe key elements of the system in more detail.

**Table 2.** Overview of registers and other data sources used in the Danish COVID-19 surveillance and vaccine monitoring system, responsible authority and type of information used

Name of data source	Responsible authority	Type of information used	Name of data source in Danish, including link
The Danish Microbiology Database (MiBa)	Statens Serum Institut (SSI)	- SARS-CoV–2 laboratory test results (positive and negative)	Den danske mikrobiologidatabase
The Danish Vaccination Register	Statens Serum Institut	- date of vaccination - vaccine manufacturer - type of vaccine - place of vaccination	Det danske vaccinationsregister
Civil Registration System (CRS)	Ministry of the Interior and Health (MoIH)	- unique civil registration number assigned to all persons residing in Denmark - age - sex - place of residence - ethnicity - household members - date of death	Det Centrale Personregister (CPR)
National Patient Register (NPR)	Ministry of the Interior and Health/ Danish Health Data Authority (DHDA)	- hospital admission - intensive care admission - comorbidities/risk factors - diagnoses during admission	Landspatientregisteret (LPR)
DREAM register^a^	Ministry of Employment/ Danish Agency for Labour Market and Recruitment	- information on type of employment	Forløbsdatabase (DREAM)
Cause of Death Register (CoDR)	Ministry of the Interior and Health/ Danish Health Data Authority (DHDA)	- date of death - cause of death	Dødsårsagsregisteret (DAR)
Authorization Register (AR)	Ministry of the Interior and Health/ Danish Patient Safety Authority (DPSA)	- identification of healthcare personnel	Autorisationsregistret
Register for Selected Chronic Diseases and Severe Mental Disorders	Ministry of the Interior and Health/ Danish Health Data Authority	- comorbidities/risk factors	Register for udvalgte kroniske sygdomme og svære psykiske lidelser (RUKS)
The Danish National Health Service Register for Primary Care (DNHSR)	Ministry of the Interior and Health/ Danish Health Data Authority	- identification of pregnant women	Sygesikringsregisteret (SSR)
The National Catalogue of Health Organisations	Ministry of the Interior and Health/ Danish Health Data Authority	- names and locations of test centres, hospitals, and hospital departments from NPR)	Sundhedsvæsenets Organisations Register (‘SOR’)
Database for Notifiable Diseases	Ministry of the Interior and Health/SSI	- travel - symptoms (only for the first 350 cases)	MIS3 – Meldesystemet for Infektiøse Sygdomme
Data from the system ‘Pandemic’	Ministry of the Interior and Health/ Danish Patient Safety Authority	- travel - symptoms - date of onset of illness - data from event-based surveillance (UEFA European Championships, etc.)	NA
‘Snapshot data’ from hospitals	Ministry of the Interior and Health/ Danish Health Data Authority	Data on COVID–19 hospital admissions are based on figures from the NPR. Since there is a certain delay in the reports to the NPR, statistics on hospitalization are supplemented with real-time data, so-called ‘snapshots’, reported daily from IT systems of the Danish regions. This constituted reporting of COVID–19 patients on a person level, as well as aggregate data of the hospital capacity, i.e. the number of occupied and available hospital beds.	NA
The Central Husbandry Register (CHR)	Ministry of Food, Agriculture and Fisheries/ Danish Veterinary and Food Administration (DVFA)	- addresses of mink farms, which were subsequently linked with addresses from the CRS and test results from MiBa to identify persons connected to mink that tested positive for SARS-CoV–2	Det Centrale HusdyrbrugsRegister (CHR)
Nursing home overview and nursing home resident database	Ministry of the Interior and Health/Danish Health Data Authority	- name of nursing home - move in date - move out date	Plejehjemsoversigten Plejehjemsadresser og plejehjemsbeboere: SDS rapport (sundhedsdatastyrelsen.dk)
List of primary schools and primary school pupils (aged 5–15 years)	Ministry of Children and Education	- school name - class	NA

a Danish acronym of ‘Den Registerbaserede Evaluering af Marginaliseringsomfanget’ translating into ‘The evaluation of marginalized groups of individuals based on registered social public transfer payments’.

**Table 3.** Overview of outputs and data deliveries of the Danish COVID-19 surveillance and vaccination monitoring system for the four major stakeholder groups

Name	Target audience^a^	Purpose and content	Format	Production/ delivery frequency	Time period of production/ operation	Danish name and Link
**(a) Information for individual citizens**						
Smitte|stop App	Citizens	Citizens could voluntarily register their positive SARS-CoV–2 test result based on PCR or antigen testing in the *smittestop* app (translating to ‘stop infection’). The app was developed to help contain the spread of COVID–19 and break chains of transmission. When an app user registered their positive test result in the app, other app users that had been in close contact with the positive case received a notification that they had been exposed to someone that tested positive, together with guidance on what to do.	Comma-separated value (CSV) file	Daily, every hour	18 June 2020–31 March 2022	www.smittestop.dk
Health web portal *Sundhed.dk* and MinSundhed app	Citizens	SARS-CoV–2 test data from the Danish Microbiology Database (MiBa), which includes results from both antigen and PCR tests, has been provided to the generic health web portal *www.sundhed.dk* that allows citizens to digitally access their own health information and their corona passport. When a person tested positive in the antigen test, they were advised to get confirmation by PCR. Only PCR-confirmed cases were included in the surveillance outputs. For SARS-CoV–2 test results, a special view was created for easier access and interpretation by citizens. As of March 2024, the special view was discontinued, and SARS-CoV–2 test results are displayed under the general section for laboratory test results.	National XML-standard for reporting of microbiology tests	Daily in real-time	View of SARS-COV–2 test results among the general laboratory test result section: ~2 March 2020 – present^g^ Special view for SARS-CoV–2 test results: 26 August 2020–7 March 2024	www.sundhed.dk Den gode XML MiBaRpport version XRPT05
Invitations for COVID–19 vaccination and reminders	Danish Health Authority (DHA)	To invite citizens 65+^b^ years of age for COVID–19 vaccination via the state digital mailbox. Persons that did not get vaccinated received reminders.	PDF file	Ad hoc before the start of the vaccination strategy	DHDA produced this output between 15 January 2021 and SSI between 15 September 2022 and 15 September 2023	NA
*Coronapas* (Corona passport, i.e. EU’s Digital COVID–19 certificate)	Citizens	SARS-CoV–2 test results from both antigen and PCR tests were visible in the corona passport via www.sundhed.dk or a separate national app, which served as a digital COVID certificate, both nationally and in the European Union (EU). It displayed either proof of vaccination, a recent infection, or a valid negative test result to facilitate safe travel and the reopening of non-essential businesses after lockdowns.	Data delivery from MiBa via the National Service platform	Daily every 15 min	28 May 2021 – 31 March 2022 (Danish part)/30 June 2023 (international part)	Coronapas-app
**(b) Data deliveries to other complementary systems and researchers**						
Prognostic mathematical models	Mathematical modelling group at SSI	To predict the evolution of the epidemic: (1) Time series of daily numbers of new positives, tested, positive percentage, and incidence by geographic units (2) Person-level data	(1) CSV-files on shared drive and website (2) CSV-files shared via a Secure File Transfer Protocol (SFTP)-server	(1) Daily/since April 2023 weekly (2) Daily	(1) 1 April 2020–16 April 2024 (2) ~1 April 2020 – present^g^	NA
Data deliveries for external researchers	External researchers	External researchers can apply for test and vaccination data through research services at the Danish Health Data Authority or Statistics Denmark (SD).	CSV-files delivered via SFTP from SSI’s Data Integration and Analysis Deparment (DIAS) to DHDA and SD.	Data was updated at DHDA daily during the pandemic. From 2022 onwards every month.	3 April 2020 – present^g^	Forskerservice - Sundhedsdatastyrelsen
Whole genome sequencing (WGS) surveillance	SSI-based Sequencing and Bioinformatics group, Virus Research and Development Laboratory	To link information from the line list with variant data of sequenced COVID–19 samples. The linked data was used for episode/case-based surveillance reports, weekly reporting to ECDC through the European Surveillance System (TESSy), sharing with WHO during biweekly meetings, as well as scientific manuscripts.	SQL Table	Daily	1 June 2021 – present^g^	Examples of outputs: https://covid19genomics.dk/statistics
Wastewater surveillance	Wastewater surveillance group at SSI	Files including the number of positive COVID–19 cases and tested persons per date and wastewater treatment plant catchment area are delivered from the automated surveillance system. The data were used to interpret wastewater results to monitor infection in the population.	CSV-files on shared drive	Daily	22 November 2021 – present^g^	NA
**(c) Information for decision-makers**						
Key Figures report	MoIH, DHA, SSI, DHDA, DPSA, National Police, Departments of Clinical Microbiology (DCMs), Danish Regions, Statistics Denmark, Modelling group, National doctors of Greenland, and the Faroe Islands	Provide decision-makers with a briefing on the latest key figures. The content included the number of persons tested in total/tested positive and vaccinated, COVID–19-related admissions and deaths, and data on hospital bed occupancy.	Automated mail including CSV-files	Once daily	20 May 2020–30 March 2023	NA
SMS service	Ministers	Key figures provided by SSI were digested in SMS form by the MoIH to provide ministers with the latest status of the epidemic on their phone.	CSV and XLSX files on shared drive	Once daily	1 March 2020–~ March 2023	NA
Case list for contact tracing	DPSA	Cases that tested positive by PCR or antigen test were shared with DPSA for contact tracing. At the start, DPSA actively contacted positive cases. On 23 December 2021, an online self-service solution for contact tracing was implemented at DPSA. After this, the case lists were used when citizens would call for additional advice.	Excel file in the beginning; later automated as an SQL table	Varying frequency over time ranging from three to six times daily	Between ~15 March 2020 and ~ 15 April 2023 (3 times daily during the first year, 4 times daily from June 2021, and gradually phased out from ~15 January 2023)	NA
Customized situation reports for Prime Minister’s Office	Prime Minister’s Office and stakeholders in other ministries	More extensive status update of the epidemic, including visualizations.	PDF file	Daily during the peak of the pandemic; weekly as of April 2023.	Produced by DHDA between May 2020 and December 2022. Production and development were taken over by SSI as of 20 December 2022–6 February 2024. An abbreviated version is still operational.	NA
Monitoring of area-specific test activity	DCSA	To monitor test activity in a specific area (i.e. municipality or parish) when new variants emerged, with the aim to test 80% of the population in that area. When the 80% coverage was reached, the production of the output was stopped.	Automated mail and CSV-files	Daily during the respective time period	Initiated for the first time when the Delta variant was detected in Denmark. It was subsequently run for short periods of time every time a new variant emerged.	NA
Report for the National Operational Staff (NOST)	NOST, MoIH, DHA, National Police, SSI, DHDA, Clinical Microbiology Laboratories, DPSA, Danish Regions	Provide the latest key figures to Denmark’s highest response group. The report contained the following indicators: - Number of persons tested for COVID–19 by test result and infection category (first time or reinfection) - Number of tests performed in total, per test site (i.e. clinical microbiology laboratories, SSI, Test Center Denmark (TCDK), and private test providers) and test type (antigen or PCR) - Overview of vaccine uptake, i.e. number of persons that received: (i) at least one vaccine dose (ii) a primary vaccination course or (iii) a booster vaccination	Automated mail	Twice daily	~8 March 2020–28 February 2023	NA
EpiMiBa report	MoH, DHA, SSI, DHDA, Regions of Denmark, DCMs	The purpose of this report is to validate data from TCDK and the DCMs as part of quality assurance (Table 2). The report is based on data from MiBa (from both public and private test providers) and the Civil Registration System (CRS). It entails summary tables with key figures of the COVID–19 epidemic by Region, test provider/ laboratory, test type, test result, sample, sampling date, age and sex, various time periods and combinations of the above.	Automated mail including xlsx-file	Ranging from five times daily in March 2020, twice daily until 28 February 2023	~8 March 2020–28 February 2023	NA
Hospital capacity report	MoIH, DHA, SSI, DHDA, DCMs, Danish Regions, DEMA	The purpose of the report was to report on the hospital capacity to, among others, DEMA, which is responsible for the planning thereof. The report contained an overview of the hospital capacity by Region, i.e. the number of hospitalized COVID–19 cases (including on intensive care and requiring mechanical ventilation) and the number of occupied and available beds by Region.	Automated mail	Twice daily	7 April 2020–13 June 2023	NA
Data deliveries for the Danish regions	Regions	SSI established data deliveries from the regions to have a real-time snapshot of the capacity in the hospitals. The same data was received from the NPR registrer but had a 24 hour delay. In return, SSI delivered these data back and added an algorithm (Table 4), so the regions had an overview of SSI’s statistics of the COVID–19 patients in their hospital.	CSV-files shared via SFTP server	Daily	1 July 2020–14 June 2023	NA
School outbreaks	MoCE, DPSA, municipalities, and the public	Number of schools with outbreaks and number of outbreaks per grade level by week and region.	Aggregated data as figures and tables at the public SSI website.	Weekly	Initially created and published by SD, with a first report published on 18 November 2020. SSI published data on its website from 4 October 2021–30 October 2022.	Report (18 November 2020): Covid–19 i Danmark. Fokusrapport: Udbrud på grundskoler (ssi.dk) Possible outbreaks in primary schools (*Mulige covid–19-udbrud på grundskoler*): Ugentlige opgørelser med overvågningsdata (archive.org) section ‘Mulige covid–19-udbrud på grundskoler’
Travel surveillance	MoIH	This output was created to monitor the travel history of COVID–19 cases.	CSV-files	Daily	~15 March 2020–13 June 2023	NA
Data delivery for status report of the Danish Health Authority	DHA	To provide data on COVID–19 cases and vaccinations for a weekly status report on COVID–19 disease burden, hospital capacity, and vaccination uptake.	CSV-files	Weekly	~15 October 2021^c^–~1 April 2023	NA
Surveillance of persons connected to mink when the first outbreaks on mink farms started in June 2020	Epidemiology Department of SSI	To provide a specific line list to merge with addresses from the CHR and CRS based on addresses and link with positive test results from MiBa to identify COVID–19 cases connected to mink farms (i.e. employees, residents, etc.). This information (a) served as an early warning for the Danish Veterinary and Food Administration (DVFA) https://en.foedevarestyrelsen.dk/ to investigate mink on the farm that was connected to a positive human case, (b) was shared with the DPSA for contact tracing, and (c) was used to flag samples from positive human cases connected to mink for WGS. Information was summarized in reports.	Linelist as an SQL table; Automated mail with PDF file	Daily (eventually weekly)	Linelist: 17 March 2020–8 February 2021 Automated mail: 16 July 2020–8 February 2021	COVID–19 i mink; see ‘*Epidemiologirapporter’*
SARS-CoV–2 surveillance in mink workers in 2023	Epidemiology department at SSI	To support a voluntary testing programme of mink workers for SARS-CoV–2 based on a temporary veterinary legislation. The objectives were (i) to investigate infection chains between mink and mink workers using WGS and (ii) to identify potential new SARS-CoV–2 variants in mink and mink workers using WGS.	SQL table; Automated mail when a positive worker was detected XLSX-file	Daily Weekly	1 January 2023–25 August 2023	NA
Automated reports for municipalities	SSI, DPSA, Municipalities	To support intersectoral meetings that assessed the need for special interventions on municipal and regional level, e.g. contact tracing, vaccination, lockdowns, etc. Reports contained time series with 7-day incidences, test rate and percent positive, vaccination uptake, and information on age and sex and country of origin. Reports were for local authorities only and were not made public.	Word and PDF files	Weekly and ad hoc as needed	As of early 2020, manual weekly reports were produced for a subset of municipalities. Between 17 June 2021 and 31 March 2022, more detailed automated weekly reports were generated for all municipalities.	NA
New cases per street for active response in municipalities	Municipalities	To support the local COVID–19 response during a time in which municipalities engaged communities by actively visiting affected neighbourhoods and streets to inform about a rise in cases. To support this activity, files with information on new cases by street were delivered each day and were closed when 80% of residents in the affected area were tested.	Excel files	Daily	25 February 2021–25 May 2021	NA
Geographic analysis by 100 × 100 m grids on incidence and vaccination coverage	Municipalities	To support municipalities with data on test activity and vaccination updates in smaller geographic areas to support local initiatives, such as increasing test activity in areas with high COVID–19 incidences and low test activity. Maps showed: COVID-incidence Test rate Vaccination-coverage	PDF maps and shape files	Weekly	11 July 2021–6 March 2022	NA
Thresholds triggering interventions by means of traffic light system	MoIH	A set of indicators with set thresholds that were used at the MoIH to monitor needs for interventions.	CSV-files on shared drive	Every Tuesday	30 September 2020–2023 March 2023	NA
Data-driven automatic local lockdowns	MoIH, relevant municipalities, citizens	A set of indicators with thresholds set by MoIH to trigger an automatic local lockdown decision at the level of municipality or parish. Based on minimal case numbers, percentage positive, and incidence (Table 4).	CSV-files on the shared drive and SSI website	Daily to MoIH at least 2 h before made public at 2 pm.	Lockdown decisions were effectuated from 12 April 2021 to 1 September 2021. SSI continued production for surveillance purposes until 28 March 2023.	Automatisk model for lokale nedlukninger
Files on breakthrough infections	MoIH, DHA, citizens	Files with information on breakthrough infections, i.e. infections after vaccination. A second version concerned only infections after booster vaccination since 15 September 2022.	CSV-files on the shared drive (later also published on the SSI website). The second version files were not made public, albeit a summary table was published in the Tendency Reports.	Once a week	First version: 10 March 2021–26 April 2022 (public from 14 September 2021) Second version: 11 October 2022–07 March 2023	Historiske covid–19-opgørelser – see ‘*Filer med overvågningsdata’*, file names ‘*gennembrudsinfektioner_tablexxx.csv’ (first version). Second version summaries under ‘Ugentlige tendenser’*
Reports on breakthrough infections	MoIH, citizens	Report containing details on breakthrough infections (with vaccine effectiveness, including as of 15 October 2021).	PDFs on the SSI website	Every 2 weeks	7 June 2021–18 January 2022	Reports on COVID–19 infections after vaccination (later reports are bilingual in both Danish and English)
Target groups for COVID–19 booster vaccinations	MoIH, DHA	To monitor vaccination uptake in target groups to monitor immunity and roll-out of the vaccination programme. Target groups were defined based on registers and algorithms. The target groups were integrated in the automated surveillance of SSI in autumn 2021. These data also contributed to estimating the number of vaccines needed to be ordered for the following season.	CSV-files, SQL-tables and data for the dashboard	Once a week	First season^d^: 15 September 2021–1 March 2022 Following seasons: 1 October–15 January	NA
EpiDVR report	MOIH, DHA, SSI, Danish Regions	To follow vaccination uptake in the population.	Automated mail	Daily	27 January 2021–30 Marts 2023	NA
Customized situation reports on vaccination for Prime Minister’s Office	Prime Minister’s Office and stakeholders in other ministries	More extensive status update of the vaccination coverage, including visualizations	PDF file	Daily during the peak of the pandemic; weekly as of April 2023	Produced by DHDA between 15 January 2021 and 13 September 2021	NA
Mortality report	MoIH, SSI, DHA	To closely monitor COVID–19 mortality: 1) among young persons 2) in general, since the start of the epidemic and the difference with the previous day	1,2) Automated mail 2) Files on the Q-drive	1) Ad hoc when death <50 years old occurred 2) Daily	1) ~15 October 2021–31 March 2023 2) 7 October 2022 – present^g^	NA
**(d) Information for the public**						
Regional Dashboard on COVID–19 infection	Citizens	The purpose was to display COVID–19 infection on a regional level. Indicators included the number of samples tested by PCR or antigen test (including total per week since the start of the epidemic), confirmed cases, first-time infections, reinfections, deaths, and new hospital admissions (including total by day since the start of the epidemic) by region and sex. Incidence/ 100.000 and percent positive over the past 7 days on regional level. Information on changes in the indicators since the previous week (previous day prior to March 2023) was phased out as of 13 September 2023.	ArcGIS Dashboard	Daily including weekends: 3 December 2020–3 June 2022 Only on business days: 3 June 2022–29 March 2023 Once a week: 29 March 2023 – present^g^	3 December 2020 – present^g^	Regionalt | Covid–19 Dashboard
Municipal Dashboard on COVID–19 infection	Citizens	The purpose was to display COVID–19 infection on a municipal level. The content included changes since the previous day in terms of the number of PCR/ antigen samples tested, confirmed cases (including over the past 7 days by age group), first-time infections and reinfections (including by sampling week since the start of the epidemic), deaths, and new hospital admissions by municipality. Incidence/100.000 and percent positive over the past 7 days on municipal level.	ArcGIS Dashboard	Once daily, including weekends: 14 September 2020–3 June 2022 Only on business days: 3 June 2022–23 March 2023	14 September 2020–23 March 2023	Kommunalt - Covid–19 Dashboard
Dashboard on Key Figures of the COVID–19 epidemic	Citizens	The purpose was to display key figures of the COVID–19 epidemic in Denmark. The content included the total number since the start of the epidemic in Denmark and changes since the previous day in terms of the number of samples tested by PCR and antigen tests, confirmed cases, first-time infections, reinfections, and deaths.	ArcGIS Dashboard	Once daily, including weekends	6 August 2020–13 September 2023	Nøgletal - Covid–19 Dashboard
Dashboard on COVID–19 Breakthrough infections	Citizens	The purpose was to monitor breakthrough infections and display the number of persons infected and hospitalized by vaccination status.	ArcGIS Dashboard	Upload frequency varied between once daily, including weekends, and only on business days	18 October 2021–25 May 2022	Dashboard om Gennembrudsinfektioner
Regional Dashboard on COVID–19 vaccination	Citizens	The purpose was to display COVID–19 vaccine uptake on a regional level. The content included an overview of the number and percentage of persons with primary vaccination courses and booster vaccinations by region, vaccine type, age group, and sex.	ArcGIS Dashboard	Upload frequency varied between once daily, including weekends, and only on business days	22 February 2021–23 March 2023	Regionalt - Covid–19 Vaccinedashboard
Municipal Dashboard on COVID–19 vaccination	Citizens	The purpose was to display COVID–19 vaccine uptake on a municipal level. The content included an overview of the number and percentage of persons with primary vaccination courses and booster vaccinations by municipality, age group, and sex.	ArcGIS Dashboard	Upload frequency varied between once daily, including weekends, and only on business days	22 February 2021–23 March 2023	Kommunalt | Covid–19 Vaccinedashboard
Dashboard on COVID–19 vaccination among target groups	Citizens	The purpose of this dashboard was to track vaccine uptake among 12 defined target groups based on occupation (e.g. social and health care workers) and other risk factors (e.g. age, nursing home residency, comorbidities, etc.). The content included an overview of the number and percentage of persons that started and completed a primary vaccination course.	ArcGIS Dashboard	Upload frequency varied between once daily, including weekends, and only on business days	19 May 2021–8 February 2022	Målgrupper | Covid–19 Vaccinedashboard
Dashboard on COVID–19 vaccination	Citizens	The purpose of this dashboard was to provide a vaccination overview among the 5 target groups for the vaccination programme of 2023/2024. The content includes an overview on vaccinations per region and shows figures of numbers and percentages of vaccines per target group, age group, and sex.	ArcGIS Dashboard	Once a week	12 October 2023–7 February 2024	Covid–19 Vaccinedashboard
Dashboard on COVID–19 immunity based on infection and vaccination	Citizens	The purpose of this dashboard was to indicate immunity in the population derived from vaccination and infection acquired as of 17 December 2021, when Omicron became the dominant variant in Denmark. In September 2022, this dashboard was archived as the protection after vaccination or infection, or a combination of both, proved to be too uncertain to calculate	ArcGIS Dashboard	Upload frequency varied between once daily including weekends and only on business days	7 April 2022–14 September 2022	Smitte og vaccine - Covid–19 Dashboard
Dashboard on COVID–19 test centres	Citizens	The purpose of this dashboard was to monitor infection during the summer, when many residents of Denmark spend time in summerhouses rather than in their primary residence. This dashboard also showed a circle with radius of 20 km around each test centre. At the time, the maximum distance from citizen to a test centre should not exceed 20 km.	ArcGIS Dashboard	Upload frequency varied between once daily, including weekends, and only on business days	1 July 2021–22 May 2022	NA
SSI website on COVID–19	Citizens	Provides general information about COVID–19, e.g. on risk groups, mode of infection, prevention, mortality, treatment and vaccination, different types of coronavirus, testing for COVID–19, the timeline of COVID–19, and other references on COVID–19, as well as hygiene advice for citizens and healthcare personnel. In addition, the website has provided access to surveillance data (including a COVID–19 tendency report and focus reports on selected topics – see below), analyses and prognoses, and lockdown model on municipality/parish levels.	Written information, graphics, and tables and downloadable zipped CSV-files	Zipped CSV-files: daily or weekly Web page graphics and tables: weekly Tendency Reports: weekly	11 November 2020 – present^g^	COVID–19 (ssi.dk) (current website) COVID–19 (archive.org)
Epidemiological Trend Reports^e^	DHA, DPSA, infection prevention and control (IPC) units at Danish hospitals, as of week 432 021 also citizens	To collate, describe, sum up, and interpret the development of the SARS-CoV–2 epidemic in all surveillance parameters (incidence, elderly care homes, hospital admissions, deaths, regions, vaccination, prevalence in the social and health section, including childcare institutions and schools). The report also included an interpretation of the current epidemiological situation.	PDF report and presentation during teleconference	Weekly report^f^ Weekly teleconference (later monthly)	29 January 2020–23 March 2023	Epidemiologisk Overvågningsrapport Historiske covid–19-opgørelser Tendensrapport Historiske covid–19-opgørelser
Risk assessments	MoIH	To provide joint risk assessments based on SSI-led epidemiological trend reports and input from the DHA on hospital capacity to the Epidemic Committee.	Editable Word documents for input by the Epidemic Committee. Later published as finalized PDFs on the dedicated Corona website.	(bi-)weekly	31 October 2020–22 February 2023	NA
Focus reports	Citizens, DHA	To describe and provide interpretation of a specific topic of interest (e.g. SARS-CoV–2 and pregnancy, schools, children, elderly care homes, ethnicity, determinants of vaccination, admissions, etc.)	PDF report made available on SSI website	Ad hoc	25 March 2020–11 December 2022	Fokusrapporter (archive.org)
Overview of waiting times for making an appointment for testing and receiving test results	(1) Citizens (2) Regions, MOIH, DHA, DCSA	Overview of waiting times in days until an appointment for testing can be booked and test results are ready after sample collection at various test centres affiliated to TCDK in the Regions. These indicators pertained to the “community track” and monitored the political decision that 80% should (i) have access to a COVID–19 test within 24 h and (ii) receive their COVID–19 test result the day after the sample collection.	(1) Figures available on the SSI website (2) Automated mail with figures and PDF files; CSV-files and figures on shared drive	Once weekly displaying data from the previous week	(1) ~31 March 2022–31 March 2023 (2) ~15 December 2020 - ~15 September 2022	NA

a For abbreviations, see Table 2.

b 50+ of age in season 2020/21.

c DHDA produced the status report before SSI took over the production.

d DHDA defined the target groups based on templates from Regions with an overview of persons eligible to receive the vaccine, before SSI took it over as of summer 2021.

e The name of this output changed during the course of the epidemic from ‘epidemiological report’, signal report from week 45, 2020 to ‘trend report’ from week 43, 2021 onwards. Trend reports became publicly available on the SSI website.

f With a few deviations from the weekly schedule during the summer months.

g Status as of end of June 2024.

#### The Danish microbiology database, EpiMiBa and Keys to Infectious Disease Surveillance (KIDS)

MiBa, as described elsewhere [5, 6], forms the core of the COVID-19 surveillance system. In short, MiBa is the national microbiology database established at SSI in 2010. It is managed by SSI’s Department for Data Integration and Analysis (DIAS), that also is responsible for the development and maintenance of the automated infectious disease surveillance system in Denmark. MiBa receives microbiology results from (i) all 10 Danish Departments of Clinical Microbiology (DCMs) across the five Regions of Denmark and (ii) reference laboratories based at SSI. MiBa serves a dual purpose: to allow healthcare providers access to patients’ microbiology test results and to provide a basis for surveillance of infectious diseases and pathogens [5, 6]. For COVID-19 surveillance, MiBa also integrated test results from Test Center Denmark (TCDK) [7], as well as PCR and antigen test results from private laboratories [5] (Figure 2).

EpiMiBa constitutes a mirrored version of MiBa, which is used as a basis for surveillance and research. For EpiMiBa, Central Dynamic Mapping is used to convert local codes submitted by DCMs into shared standard coding and uniform terminology to facilitate data extraction and statistical analysis [6].

Raw data from EpiMiBa is subsequently standardized in the KIDS, where the following levels are applied to the data through the use of algorithms: (1) demarcation of all potentially relevant test reports, that is all test reports with SARS-CoV-2 mentioned in their requisition or results codes, (2) interpretation of test reports (i.e. categorization into positive, negative, or irrelevant results for SARS-CoV-2) and application of the so-called ‘patient-date’ (Table 4), and (3) application of episode definition, where test results are converted to cases and are used to determine reinfections (i.e. time window of 60 days between two SARS-CoV-2 infections) (Table 4) [8].

**Table 4.** Overview of algorithms used in the Danish COVID-19 surveillance and vaccination monitoring system

Name of algorithm	Purpose	Definition	Data sources used^a^	Duration of application
**Applied *before* linking the line list with other data sources**				
Reinfections	To monitor the burden and development of reinfections, to register admissions and deaths related to reinfections, and to define metadata at the time of reinfection, e.g. age, occupation (i.e. healthcare workers), pregnancy, and other risk factors	A person is included in the COVID–19 surveillance system as of the timepoint of the first SARS-CoV–2 test. The first positive test result represents the onset of an episode. If the same person has another positive test more than 60 days after the onset of the preceding episode, a person is considered to have a reinfection. It is not a requirement to have a negative test between the two positive tests, and there is no limit as to how many reinfection-episodes a person can have. Any positive tests within 60 days after an episode onset are not considered a start of a new episode.	MiBa	13 December 2021– present^c^
**Applied *during* linking of line list with other data sources**				
COVID–19-related hospital admissions	To monitor the number of COVD–19-related hospital admissions	A COVID–19-related hospital admission is defined as an admission of a patient within 14 days after the sampling date of the first positive SARS-CoV–2 test result. Reinfections are included in COVID–19-related admissions if admitted within 14 days of the positive test result. Persons that test positive for SARS-CoV–2 while being hospitalized are also registered as COVID–19-related hospital admissions. If the person tests positive for SARS-CoV–2 more than 48 h after the admission date, the COVID–19-related admission the test date instead of the admission date is used. Hospitalizations include patients who have been registered in at least one snapshot or who, according to the NPR, are or have been hospitalized for more than 12 h. Intensive care admissions that last less than 12 h are also included. Short hospitalizations are defined as <12 h in the snapshot data. Patients who have been admitted to the intensive care unit for <12 h, and patients who died within 12 h of admission are not included in the short admissions.	MiBa NPR Snapshot data	Covid–19 related admissions were included in the epidemiological report from 17 of March 2020. Snapshots were automatically reported from 7 April 2020. Snapshot data were phased out from surveillance data on 13 June 2023. Before 7 April 2020 and after 13 June 2023 until present, the algorithm has solely been based on the National Patient Registry.
Patient date	To organize data to ensure consistent and fast calculations	If a person has undergone multiple SARS-CoV–2 tests per day, only one test per day is kept for calculations. We defined an algorithm based on a hierarchy to select the most important (i.e. reliable) test. A variable indicates when a test has to be included in the numerator and the denominator, which allowed equal calculation of, e.g. positive percentage across surveillance outputs.	MiBa	May 2020 – present^c^
Admissions *with* or *due to* COVID–19	To distinguish hospital admissions due to or with COVID–19	As new variants came to Denmark, an alternative algorithm to COVID–19 hospital admissions (see above) was developed. Instead of using time from the sample date, we estimated admissions *due to* COVID–19 by using diagnosis codes from the NPR.	MiBa NPR	First published on 6 January 2022 and revised on 10 August 2022
End of acute infection	To monitor how many persons have overcome acute infection, i.e. recovered	A patient must meet at least one of the following criteria to be classified as having overcome an acute COVID–19 infection: • When a person is not admitted within 14 days of the test date, the end of the infection date is day 14. • When a person is admitted to a hospital within 14 days of the test date and discharged again within 14 days, the date of end of acute infection is 14 days after the test date. • When a person is admitted within 14 days of the test date and discharged between days 14–30 from the test date, the date of end of infection is the discharge date. • When a person is hospitalized within 14 days of the test date and is still hospitalized on day 30 (in a non-intensive care unit), the date of end of infection is day 30 at the latest. • When a person is admitted within 14 days of the sample date and is still admitted to the intensive care unit on day 30, the date of end of infection is the discharge date from the intensive care unit, however a maximum of 90 days after the sample collection date.	MiBa NPR CPR CoDR	Relevant at the start of the pandemic. First published on 1 April 2020. Phased out on 13 June 2023
30-day mortality	To monitor mortality	If a patient dies within 30 days of testing PCR-positive for SARS-CoV–2, the patient will be counted as a COVID–19-related death. The civil registration number (CPR) register is updated daily, but recording of death can have a delay of up to 3 days. The CPR register is not updated outside working days. The Cause of Death Register is more timely and is updated several times a day, including on weekends and during holidays. Therefore, the date of death from the CPR register and the Cause of Death Register were combined to calculate the 30 days mortality. From 12 to 28 March 2020, a 60-day mortality was reported based on experience with SARS-CoV–1 that showed that patients can be admitted to the hospital for up to 90 days before dying. After dialogue with ECDC, we adjusted it to 30 days to be more comparable to other countries and have a more specific definition.	CPR register CoDR	60-day mortality from 12 March 2020 to 28 March 2020 30-day mortality from 29 March – present^c^
ICU admissions	To monitor ICU admissions	The National Catalogue of Health Organisations’ procedure codes from NPR are used to identify Intensive Care Treatment (ICT), such as intensive care therapy or observation, respirator use, need for ventilation, Extracorporeal Membrane Oxygenation (ECMO) or treatment for heart conditions. Data were linked to the patient linelist and data from the Danish Microbiology Database (MiBa) to keep patients that tested positive for COVID–19. Data is kept, if the procedure code occurs during the same course of admission (14 days from the sample date)	NPR MiBa	15 April 2020 – present^c^
Pregnancy	To identify pregnant COVID–19 cases	A person is defined as pregnant if: the sex is female the age on the sampling date is between 15 and 45 and if one of the following criteria is fulfilled: in the 30 weeks before the sampling date, the person is registered in the DNHSR or NPR with at least one pregnancy-related code and is not registered with a code for birth/abortion (exception: if a pregnancy-related code is registered more than 4 weeks after the last birth/abortion code, then the person is considered pregnant) the person is registered with one of the codes for birth/ abortion in the 4 weeks after the sampling date	NPR DNHSR	4 April 2020 – present^c^
Comorbidities	To monitor cases with co-morbidities	A person is defined to have one or more comorbidities if the person has one or more of selected diagnosis codes within 5 years before the sampling date.	NPR, Register for Selected Chronic Diseases and Severe Mental Disorders	3 April 2020 – present^c^
**Applied *after* linking the line list with other data sources and algorithms developed based on line list**				
Death *with* or *due to* COVID–19	To monitor mortality attributed to COVID–19 vs. other causes	Death with or due to COVID–19 was distinguished based on two approaches [4]: 1. Probability calculations based on PCR test activity, test positives, and overall mortality, by week and age group: 2. Underlying Cause of Death on validated death certificates of COVID–19-related deaths	1. Data from MiBa and CRS 2. Live validated Cause of Death Register, aggregated delivery from DHDA	1. 14 July 2022–21 March 2023 2. 11 February 2022–30 March 2023
Reproductive number (Rt)	To monitor the evolution of the epidemic	The Rt is an expression of an infectious disease’s ability to spread in the population. Rt describes how many people an infected person will infect on average in a population in which some individuals are protected against the disease. The Rt depends on the number of contacts one has, the risk of transmission of infection with each contact, and the duration of the infectious period. These factors can be influenced by various measures. If Rt < 1, the epidemic will eventually subside. At Rt > 1, the epidemic will grow. Rt therefore gives an indication of whether an epidemic is growing, holding steady, or shrinking. See [5] for statistical details.	MiBa	15 December 2020–7 February 2023^b^
Breakthrough infections	To monitor the number and incidence of cases by severity after completed primary vaccination or booster vaccination	A breakthrough infection is defined as an infection that occurs after the expected full effect of the primary vaccination course or a booster vaccination. Vaccine uptake status as of the timepoint of a positive SARS-CoV–2 test defined the groups used for weekly counts and incidences of cases by severity (i.e. subsequent new admissions to hospital or ICU, or deaths). Test rates per group were also measured. Considering the risk-time for each person at each stage of the vaccination course (unvaccinated, vaccine dose 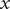 , full effect after dose 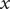 ), the algorithm keeps tabs on the changing background population of uninfected persons as denominators in the incidence calculations (counts per 100 000 person-weeks). The population was stratified by age groups. Two versions were in operation: The first version focused on the first infection and the primary vaccination course. The second version was introduced to accommodate reinfections as included in the COVID–19 surveillance system and focused on breakthrough infections only in relation to booster vaccination since 15 September 2022. The second version also computed proportions of new hospital admissions by vaccine status and age. A simplified algorithm of the dashboard was used for the Dashboard on COVID–19 Breakthrough infections (Table 3).	MiBa and DVR	First version: 10 March 2021–26 April 2022 Second version: 11 October 2022–07 March 2023
Criteria for data-driven automated lockdowns on parish and municipality level	To guide when local lockdowns should be initiated	The criteria for data-driven automatic local lockdowns at parish levels were based on three indicators measured over the past 7 days: Incidence of COVID–19 cases per 100 000 citizens Minimal number of COVID–19 cases in total The percentage positive For municipalities, the criteria were initially based only on incidence and later also minimum case numbers. The parish or the whole municipality could reopen again when one of the indicators was below the threshold for a week in a row. DPSA, in collaboration with the municipality, evaluated for quicker reopening of selected institutions.	MiBA, CRS for current population data per municipality or parish	Automated local lockdown from 12 April 2021–1 September 2021. After that, the model continued until 28 January 2022, but only for recommended measures.
Distance from test centres	To assess the maximum distance from a citizen’s residence to a test centre to ensure easy access to testing	The direct line distance was used for simplicity reasons rather than the driving distance via road.	DCSA provided geographic coordinates of locations of test centres	1 July 2021–22 May 2022

a See Table 2 for details.

b Period of automated production; manually calculated as of July 2020.

c Status as of end of June 2024.

SARS-CoV-2 test results formed the core of the surveillance system, which was therefore heavily influenced by the test strategy and capacity. In addition to the public testing system as described elsewhere [5, 9], private vendors provided SARS-CoV-2 PCR and antigen tests as of summer 2020. These became publicly funded at the end of 2020 to expand the free-of-charge test capacity. By March 2021, test results from private providers, including antigenic tests, were fully integrated in the surveillance system and were used for screening, contact tracing, and the Digital COVID-19 certificate (Figure 1a, Table 3).

#### The Danish vaccination register (DVR) and EpiDVR

The DVR was implemented in 2012 to support monitoring of vaccination coverage and estimating vaccine effectiveness. It is based on mandatory registration of all administered vaccinations, including those not included in the national vaccination programme [10].

EpiDVR is a mirrored version of DVR used for monitoring vaccination coverage and the childhood vaccination programme. This system includes a function for sending out electronic invitations to vaccination appointments, reminders before appointments, and reminders if the recommended vaccination time is exceeded by 4 weeks.

The roll-out of the COVID-19 vaccination programme started on 27 December 2020 and coincided with the second national lockdown (Figure 1a). SSI, together with the Danish Health Data Authority (DHDA), developed a monitoring system to track COVID-19 vaccine uptake, coverage, and effectiveness, as well as the capacity of vaccination centres.

#### Line lists, data sources, data linkage and algorithms

Standardized test results have been subsequently linked with data from national registers and other data sources through CPR number to create a line list. A line list is a table with key information, where each row represents a person and each column represents metadata, such as demographics, clinical and epidemiological information. Dedicated surveillance and vaccination line lists have formed the basis for the majority of outputs described hereafter (Table 3).

Table 2 shows an overview of the data sources, the responsible authorities, and including descriptions of the types of data used from the data sources. Before, during, and after data linkage, various algorithms have been applied (Table 4). These vary in complexity and consist of business rules used for automation processes. Each algorithm was developed and documented by a multidisciplinary team using available international literature and guidelines, as well as clinical expertise. Algorithms were approved on director’s level and in some cases ministerial level. The algorithms were validated with the available means, such as clinical data. Where validation based on clinical data was not possible, algorithms were validated through thorough data analysis, investigating outliers, and scientific discussions. Later in the epidemic, an algorithm for reinfections was developed, and the line list was changed to represent episodes of infection rather than unique persons. Documentation on the algorithms and limitations for interpretation was published on SSI’s website.

## Results

### Cohort included in the COVID-19 surveillance and vaccination monitoring system

Based on the Civil Registration System (CRS) (Table 2), the Danish population counts ~5.9 million by 2023. Differences in testing patterns during the three epidemic waves of SARS-CoV-2 in Denmark by demography have been described elsewhere [9]. Confirmed cases were registered based on their first SARS-CoV-2 PCR test. As of 27 June 2024, 3 438 887 COVID-19 cases, including reinfections, and 9863 COVID-19-related deaths have been recorded since the beginning of the epidemic in Denmark.

To track vaccine uptake, the vaccination monitoring system comprised the entire population of Denmark. Since the start of the vaccine roll-out, 4 737 718 persons have received at least one vaccination.

### Outputs and use of data by various stakeholders

Outputs from the automated surveillance system can be divided into four stakeholder categories listed below. Table 3 shows an overview of outputs by stakeholder group, name, target audience, purpose, content, frequency, and time period of production and links where available. Figure 1b shows when various outputs were produced in relation to the COVID-19 epidemic in Denmark.

#### Information for individual citizens

Information for individual citizens (Table 3a) entailed online access to their own SARS-CoV-2 test results and their COVID-19 vaccinations through a generic web portal *sundhed.dk*, including the corresponding app *MinSundhed* (‘MyHealth’). This platform, which already existed before the COVID-19 epidemic, provides access to any kind of diagnostic test result from all clinical laboratory-specialities, vaccinations, and other health information. Displayed microbiological test reports are obtained from MiBa (Figure 2). Physicians can access the same information through a patient’s electronic health record [5].

Analysed data from KIDS were automatically provided to the Digital COVID-19 Certificate (in Danish: *Coronapas* App) that documented vaccination, previous infection, or recent negative test results. It offered a ‘two-in-one solution’: (a) the *International Coronapas* was mandatory for travelling abroad [11] and (b) the *Domestic Coronaspas –* based on automatic business rules – was mandatory for entering events in Denmark. Large-scale testing and a fast turnaround (15–20 min from the test result was available at the laboratory until the *Coronapas* was automatically updated) contributed – among others – to keeping society as open and safe as possible. KIDS data was also fed to the *Smitte|stop* app (‘transmission stop’) that was used to inform citizens if they had had close contact with a SARS-CoV-2-positive person.

Also, in 2020, DVR’s electronic invitation-functionality was expanded to send invitations to citizens in the target groups for COVID-19 vaccination.

#### Data deliveries to complementary systems and researchers

In April 2020, the Ministry of the Interior and Health (MoIH) tasked SSI with forming an expert group for mathematical modelling, which has since received data from the surveillance system. The purpose was to support predictions about the evolution of the epidemic in Denmark, the burden on hospitals, and regional predictions for the number of new COVID-19 cases, including the effect of actions for gradual reopening after the lockdowns [12].

Other surveillance applications that used data from the surveillance system included whole genome sequencing (WGS) surveillance and wastewater surveillance (Figure 2). For WGS surveillance, data from the line list was linked to sequencing results. For wastewater surveillance, the surveillance system provided information on the daily PCR testing rate and incidence within the catchment area of each wastewater treatment plant [13]. Researchers have also had the opportunity to request daily updated data through dedicated application processes set up by DIAS and the DHDA (Tables 1 and 3b).

#### Information provided to decision makers

Recipients of data deliveries included SSI-internal and external national and international stakeholders (Table 3c).

Data for decision-makers was provided in various ways, for example as automated e-mails and comma-separated value (CSV) files that included, among others, key numbers for daily meetings on preparedness and hospital capacity planning. Data were provided close to real-time and multiple times daily, which also required rapid epidemiological interpretation by SSI-internal stakeholders.

At the MoIH, this information was converted into an SMS-service to ensure continuous status updates for ministers. In addition, the Prime Minister’s office required a more extensive PDF file with visualizations of the current status.

The Danish Patient Safety Authority (DPSA) received data on new PCR and antigen test-positive persons, including key metadata for contact tracing (Table 1).

Regions and municipalities formed a new group of stakeholders during the pandemic. Several outputs were developed to directly support the regional and local level in managing the COVID-19 response (Table 3c).

#### Information for the public

For the public, SSI developed various interactive COVID-19 dashboards (Table 3d), among others, for infection and vaccination on a regional and municipal level, as well as a dashboard with key figures of the epidemic. Between August 2020 and March 2023, the dashboards were updated daily. Prior to uploading, underlying data from the dashboards were shared with authorities under embargo. By April 2023, when COVID-19 was no longer considered a disease of societal concern in Denmark, the frequency of updating the regional dashboard decreased to once a week, and vaccine dashboards were archived but still remain accessible. For the COVID-19 vaccination programme of 2023/2024, a new vaccine dashboard was launched. CSV-files with data underlying the dashboards and other supplementary calculations were uploaded daily until 29 March 2023. Afterwards, the frequency of data uploads had been reduced to weekly. In addition, SSI published data on its website in the form of figures and tables once a week (Table 3d).

## Discussion

This article describes how the Danish COVID-19 surveillance and vaccine monitoring system was built and how key outputs were used to support the epidemic response of various stakeholders. The emergence of the COVID-19 epidemic constituted the first time in Denmark that close to real-time infectious disease data were provided to decision-makers and the public [5]. Data on (re)infection and vaccination not only guided the national COVID-19 response but also supported interventions and outbreak investigations in special settings, for example long-term care facilities [14], schools [15], mink farms [16], and large-scale events, such as the European Soccer Championships [17]. Data also enabled addressing public health research questions, for example to investigate SARS-CoV-2 transmission within households [18], protection against reinfection after primary infection [8, 19], risk of hospitalizations associated with different SARS-CoV-2 variants [20, 21], COVID-19 vaccine effectiveness and uptake [22, 23], and long COVID [24].

### Building on existing foundation

Denmark is one of the internationally leading countries with respect to establishing integrated digital solutions in the health sector [25], and earlier investments in digitalization in the health field have laid a solid basis for surveillance. Before SARS-CoV-2 emerged in Denmark, the prerequisites for real-time, automated reporting, including MiBa and DVR, had been in place for more than a decade. This allowed rapid integration and upscaling of surveillance for COVID-19. The system has proven highly flexible and adaptable, was supported by a high test capacity and a low threshold to access testing, and covered the entire population of Denmark. Having had the infrastructure in place to accommodate the strategy of mass testing and to digitally handle the associated high amounts of real-time data can be considered a major achievement in infectious disease surveillance. Although developed under high time pressure, the system proved stable and largely ran effectively throughout the pandemic. System breakdowns were rapidly addressed and therefore resulted in very limited downtime.

The availability of the population-wide number of tests performed allowed representative outputs, e.g. calculation of percent positive and the reproductive number (R_t_). The high degree of completeness (i.e. all data on tests, vaccinations, and hospital admissions were captured), accuracy, and timeliness in both the numerator and denominator was, among others, a prerequisite to enable local responses, for instance, for setting sensitive data-thresholds to initiate lockdowns on a parish level that were linked with high political, social, and economic consequences [26].

### Transparency and data sharing

In Denmark, there is a high level of trust between authorities and the public [27], which has been conducive to compliance with the testing and vaccination system. Data were continuously shared with authorities and researchers throughout the epidemic. Data shared on the SSI website were used by the media and citizen scientists for customized visualizations. In addition, SSI actively addressed questions by the media and the public on the SSI website and social media.

The availability of timely data had also sparked an interest of various national and international stakeholders and societies, which led to a substantial increase in requests for data and access to documents. To support SSI, the DHDA assisted with selected data deliveries. To ensure close collaboration regarding surveillance data between authorities under the MoIH, a data coordination group was established, which was chaired by SSI. The group included representatives of MoIH, the Danish Health Authority (DHA), DPSA, and DHDA (Table 1).

### Human resource challenges and organizational considerations

Human resources were an important aspect that challenged the automated COVID-19 surveillance. The close-to-real-time access to data and high level of transparency required tailoring outputs for multiple stakeholders with competing timelines and priorities, responding to ad hoc requests, and predicting the needs of decision-makers. In addition, epidemiological interpretation of outputs and communication had to be pursued under high time pressure and political and media attention. To meet the demand, SSI expanded significantly in the course of the epidemic. Onboarding new staff, coordinating across fast-growing departments and recruiting data scientists posed particular challenges. In 2017, SSI established DIAS, a multidisciplinary department encompassing specialists in microbiology, epidemiology, IT architecture, data science and engineering, project leadership, and communication to facilitate the needed crosscutting working environment for the development and management of these systems and enable in-depth collaboration with other departments at SSI.

### Streamlining operation of the surveillance system

As the COVID-19 systems became more complex, incident- and change-management processes were introduced to further streamline maintenance, development, and communication with users. In addition, duty rosters were implemented. Since the start of the epidemic, the following teams with different capabilities have been on duty simultaneously to ensure the functioning of the system: incident management, data management, IT-infrastructure, and leadership. Since January 2023, the weekend duties have been discontinued, retaining a minimal run of the surveillance system to display only key figures. In April 2023, further downscaling took place.

### Legal challenges

Data sharing and legal aspects also posed challenges. Despite guidance through the General Data Protection Regulation (GDPR) [28], various issues had to be evaluated on a case-by-case basis, and national legislation changed several times according to the assessed level of threat SARS-CoV-2 posed to society. This influenced the COVID-19 surveillance system, both in terms of the legal basis for receiving and sharing data as a health authority and in terms of the GDPR. For instance, outputs with personally identifiable information (PII) needed to be further aggregated or blacked out, when necessary. However, as information sharing with the public in the context of a high threat was important, SSI considered it proportionate on a case-by-case basis to show PII in selected outputs, for example showing the number of deaths in younger age groups or low vaccination coverage in a specific area and age group. Every time legislation changed, case-by-case judgements needed to be re-evaluated. An additional challenge was that authorities under the MoIH constitute separate legal entities with separate legal permissions for data sharing. This meant that data sharing between authorities under the MoIH was more complicated than sharing solely within SSI, as the interpretation of the Data Protection Act [29] – in terms of what is considered PII in aggregate data – differed between authorities. In addition, the legal basis for data sharing with certain stakeholders was not yet established for some authorities. For instance, SSI was not allowed to directly share certain outputs with municipalities. Consequently, data had to be sent via the DPSA, which had the required legal basis to share.

### Limitations of existing data sources and IT infrastructure

Denmark has a long history of high-quality national registers that proved valuable during the COVID-19 epidemic. However, the epidemic also highlighted some gaps. For instance, there was no register of nursing homes and nursing home residents or schools and school pupils available for the health authorities. This required temporary solutions specifically developed for the surveillance of COVID-19. In case of a new threat, these solutions would need to be reinstated. Similarly, a register of residences for vulnerable groups, such as psychiatric patients, was lacking. Developing new registers for these groups would be beneficial to more effectively monitor infections in these populations in the future.

Varying timeliness between the different data sources posed another challenge. For instance, MiBa provides real-time data, whereas the National Patient Register (NPR; Table 2) – providing information on hospitalizations – has a delay of a few days varying by region. As this challenges timely monitoring of and responding to fast-spreading diseases, improving the timeliness of the NPR is recommended. During the COVID-19 epidemic, this current limitation was solved by setting up a new and temporary active reporting-system, the so-called ‘snapshot-data’ (Table 2). Through this solution, the Danish Regions reported twice daily. Also, the CRS (Table 2) – from which, among others, the date of death was extracted – is not updated on weekends. To address this limitation, an additional connection was established to the Cause of Death Register (CoDR) (Table 2), which records the date of death also on weekends. The dynamic nature of real-time data from MiBa was dealt with by agreeing to freeze data at set time points per day to ensure matching figures between outputs. Another challenge was a lack of server capacity. Upscaling SARS-CoV-2 surveillance was achieved by migrating servers at the expense of other surveillance systems that needed to be shut down at the time. Additional human and financial resources are needed to ensure a stable and flexible surveillance system capable of monitoring multiple threats concomitantly.

### Aspects influencing the data

Data entering the surveillance system depended on the test strategy. At the same time, data *from* the surveillance system formed the basis for decision-making and developing the strategy. Due to this interdependence, data needed to be explained and interpreted with caution. For instance, a decrease in incidence did not necessarily reflect decreasing transmission, but a change in the test strategy or test seeking behaviour of the population influenced by information from the government or media. We also observed an interplay between data and behaviour in the effective reproductive number Rt (Table 4), which describes how many persons – in a population where some individuals are protected against the disease – an infectious person on average can infect altogether during their infectious period. Anecdotal evidence suggested that some people tended to use the Rt to judge whether it could be regarded as safe to attend public events.

The outputs from the surveillance system have been largely driven by ministerial requests. This often required navigating the interface between public health and politics, as the decision-making process during the epidemic response also took political, economic, and social interests into account. At times this resulted in challenges regarding prioritization of requests and development of meaningful algorithms and calculations.

## Conclusions

To conclude, the COVID-19 epidemic constituted a pressure test that led to useful insights regarding the possibilities and limitations of the system, as well as valuable learning points for the future. It confirmed that future surveillance systems need to be (i) sufficiently agile for rapid up- and downscaling and responding to newly emerging health threats, (ii) require sustainable financial investments, and (iii) depend on the attraction and retention of highly specialized staff, including data science talent [30].

The strong digital data infrastructure allowed stakeholders to navigate the COVID-19 epidemic based on high-quality, transparent data. In line with the goal of the EU4Health Programme that supports EU Member States in the implementation of digitalised, integrated surveillance systems [31], several developments are ongoing to further improve and prepare for future threats, for example the integration of standardized conclusions from WGS into MiBa and algorithm-based interpretation of MiBa data to facilitate automated linkage with other data sources to achieve truly integrated surveillance.

This surveillance system constitutes a model for the future infectious disease surveillance system in Denmark, including surveillance of more than 80 infections and resistant microorganisms. Denmark is in the process of a digital transformation, which not only includes technical developments and targeted data outputs but also changes in workflows, responsibilities, and organization. This process will draw on experiences gained from the COVID-19 epidemic and will build on collaborations with authorities and other stakeholders established over the past years, as effective prevention and control of infectious diseases is dependent on strong intersectoral partnerships. With the current pandemic threat posed by avian influenza viruses and the global concern of antimicrobial resistance, lessons learnt from the COVID-19 pandemic as described here still remain topical and relevant [32, 33].
