# Data for action – description of the automated COVID-19 surveillance system in Denmark and lessons learnt, January 2020 to June 2024 – CORRIGENDUM

**DOI:** 10.1017/S095026882510006X

**Published:** 2025-05-23

**Authors:** Gudrun Witteveen-Freidl, Karina Lauenborg Møller, Marianne Voldstedlund, Sophie Gubbels, Statens Serum Institut COVID-19 Automated Surveillance Group

**Affiliations:** 1 Department of Data Integration and Analysis, Infectious Disease Preparedness, Statens Serum Institut, Copenhagen, Denmark; 2 Infectious Disease Epidemiology and Prevention, Infectious Disease Preparedness, Statens Serum Institut, Copenhagen, Denmark; 3 Infectious Disease Preparedness, Statens Serum Institut, Copenhagen, Denmark

When this article was originally published in Epidemiology and Infection it contained a punctuation error in the abstract. It also contained an error in the name of the author for reference 26. These have both been updated and corrected.
